# Evaluation of Measured Resting Metabolic Rate for Dietary Prescription in Ageing Adults with Overweight and Adiposity-Based Chronic Disease

**DOI:** 10.3390/nu13041229

**Published:** 2021-04-08

**Authors:** Ciara Cooney, Ed Daly, Maria McDonagh, Lisa Ryan

**Affiliations:** Department of Sport, Exercise and Nutrition, School of Science and Computing, Galway-Mayo Institute of Technology, Galway Campus, Dublin Road, H91 T8NW Galway, Ireland; ciara.cooney@gmit.ie (C.C.); ed.daly@gmit.ie (E.D.); Maria.McDonagh@gmit.ie (M.M.)

**Keywords:** resting metabolic rate, prediction equation, ageing adults, overweight, obesity, adiposity-based chronic disease, energy balance, metabolism

## Abstract

The primary objective of this study was to compare weight changes in two groups of ageing Irish adults with overweight and adiposity-based chronic disease: participants who had dietary energy requirements prescribed on the base of measured RMR and participants whose RMR was estimated by a prediction equation. Fifty-four Caucasian adults (male *n* = 25; female *n* = 29, age 57.5 ± 6.3 years, weight 90.3 ± 15.1 kg, height 171.5 ± 9.5 cm, BMI 30.7 ± 4.6 kg/m^2^) were randomly assigned to a dietary intervention with energy prescription based on either measured RMR or estimated RMR. RMR was measured by indirect calorimetry after an overnight fast and predicted values were determined by the Mifflin et al. (1990) prediction equation. All participants received individual nutritional counselling, motivational interviewing and educational material. Anthropometric variables, blood pressure, blood glucose and blood lipid profile were assessed over 12 weeks. Body weight at week 12 was significantly lower (*p* < 0.05) for both groups following dietary interventions, mRMR: −4.2%; eRMR: −3.2% of initial body weight. There was no significant difference in weight loss between groups. Overall, 20.8% mRMR and 17.4% of eRMR participants experienced clinically meaningful (i.e., ≥5% of initial weight) weight reduction. Weight reduction in adults aged ≥50 years over the short term (12 weeks) favoured a reduction in blood pressure, triglycerides and glucose, thus reducing cardiovascular disease risk factors. This research indicates that employing a reduced-calorie diet using indirect calorimetry to determine energy needs when improving weight outcomes in adults (>50 years) with overweight and adiposity-based chronic disease is equal to employing a reduced-calorie diet based on the Mifflin et al. (1990) prediction equation. A reduced-energy diet based on mRMR or eRMR facilitates clinically meaningful weight reduction in adults (≥50 years) over the short term (12 weeks) and favours a reduction in blood pressure, triglycerides and glucose, thus reducing cardiovascular disease risk factors. Moreover, the addition of motivational interviewing and behaviour change techniques that support and encourage small behaviour changes is effective in short-term weight management.

## 1. Introduction

The high prevalence of overweight (defined as a body mass index [BMI] ≥ 25 kg/m^2^) and obesity (defined as a BMI ≥ 30 kg/m^2^) among older Irish adults is a major health concern. Obesity is an ‘adiposity-based chronic disease’ (ABCD) that affects 35% of Irish adults aged 50 years and over, with a further 44% classified as overweight [1]. ABCD is associated with an increased risk of cardiovascular diseases (CVD), osteoarthritis, type 2 diabetes mellitus and impaired functionality [2,3]. Furthermore, older adults with ABCD are reported to be at greater risk of depression, disability and frailty than their age-matched counterparts of normal weight [1]. Central obesity, which affects over half (53%) of older Irish adults, is characterised by increased abdominal adiposity and is associated with a greater risk of adverse metabolic and cardiovascular outcomes than overall obesity [4]. In addition, a higher prevalence of diabetes, high blood pressure and cardiac events was reported in older Irish adults with increased waist circumference (WC) and BMI than adults with normal WC and BMI [1].

Clinical guidelines available for the management and treatment of adults with obesity recommend lifestyle interventions involving diet, physical activity and behaviour modification for conventional obesity, with pharmacotherapy and surgical intervention for severe obesity cases [5,6,7]. Strategies to prevent weight gain, optimise weight loss and achieve long-term weight loss maintenance remain the hallmark of overweight and obesity treatment and management guidelines. Lifestyle weight management programmes consisting of reduced energy intake via calorie-restriction strategies and increased energy expenditure through increased physical activity are recommended with the support of a multidisciplinary team of health care professionals [6]. Calorie-restriction strategies such as low-calorie diets (LCD; 800–1600 kcal daily) may not be nutritionally complete and have long-term low compliance, and very low-calorie diets (VLCD < 800 kcal daily) require medical supervision due to the increased risk of medical complications [8]. For sustainable weight reduction the National Institute for Health and Care Excellence (NICE) recommend dietary approaches that reduce calories by 600 kcal/day, i.e., 600 kcal less than the individual requires to remain the same weight [9]. Similarly, a modest reduction in energy intake (500–750 kcal/day) is recommended for older adults by The American Society for Nutrition, the North American Association for the Study of Obesity (NAASO) and The Obesity Society [8]. In order to determine individual energy requirements, an assessment of resting metabolic rate (RMR) is recommended [10]. RMR is the main component of energy expenditure and accounts for up to 70% of total daily energy expenditure (TDEE) with the thermic effect of food and physical activity accounting for 10% and 20%, respectively. Energy expenditure associated with physical activity may be subdivided into energy utilised specifically for exercise, and non-exercise activity thermogenesis (NEAT) which involves maintaining posture and fidgeting [11]. RMR can be measured (mRMR) by respiratory indirect calorimetry (IC) or estimated by prediction equations (eRMR) [12]. IC is considered to be an accurate method of determining RMR [13]. IC is based on the indirect measure of the heat expended by nutrient oxidation, which is estimated by monitoring gas exchange, i.e., the volume of oxygen consumption (VO_2_) and carbon dioxide (VCO_2_) production over a period of time [13]. Such measures also provide information on energy substrate utilisation. The ratio of CO_2_ production to O_2_ consumption is known as the respiratory exchange ratio (RER) and represents fuel oxidation by IC [14]. During carbohydrate oxidation, there is an equal amount of CO_2_ produced for O_2_ consumed (RER = 1.0). During fat oxidation, there is less CO_2_ produced for O_2_ consumed [14]. A greater fractional oxidisation of fat (FAT-OX) as fuel is important for metabolic health, weight management, and body composition [15,16]. For instance, the skeletal muscle of adults with obesity, or insulin resistance displays an impaired ability to oxidise fat [17,18,19,20]. Decreases in skeletal muscle metabolic activity are associated with the ageing process and closely linked to age-related loss of muscle mass [20]. In addition, a high RER, which is indicative of low FAT-OX relative to carbohydrate oxidation, is predictive of both future body mass gain and fat mass (FM) regain after diet-induced reductions in body mass [21]. This information may be of particular relevance to ageing adults, particularly adults aged 50 years or older as a higher fat mass in relation to body mass accelerated the decline of muscle quality in this population [22]. Therefore, being able to accurately measure a person’s ability to oxidise fat can have important implications for dietary manipulation strategies and may be more important in this age group.

IC has high reproducibility and is non-invasive; however, its use outside of clinical care settings is limited with commonly cited reasons including device expense, time required to carry out the measure, and the need for trained technicians to operate equipment and interpret test results [23]. Several metabolic rate prediction equations have been developed to calculate RMR and are frequently adopted by health care professionals to determine energy needs in order to develop nutritional support plans. The primary components used to develop prediction equations include weight, height, age, sex and body composition parameters [24]. Great variability has been reported in the accuracy of RMR prediction equations employed in adults with higher than normal BMIs, often resulting in the under- or over-estimation of an individual’s specific calorie needs [25]. This may be because equations used were developed for a specific cohort such as normal weight individuals, whose characteristics differ from this population [26]. A minority of studies have validated prediction equations in adults with higher BMIs [27]. Moreover, the Mifflin et al. [28] prediction equation has been shown to provide a reliable estimate (78% within ± 10% limit of actual) of RMR in adults with normal weight and obesity [29,30]. Where IC is not available, the American Dietetic Association (ADA) recommend using the Mifflin et al. [28] prediction equation using actual body weight—males: 10 × weight (kg) + 6.25 × height (cm) − 5 × age (years) + 5; females: 10 × weight (kg) + 6.25 × height (cm) − 5 × age (years) − 161 to estimate RMR (eRMR) in adults with overweight or obesity [29].

A gradual decline in RMR and TDEE is associated with advancing age, diminished lean mass, energy restriction and weight loss [8,31,32,33]. Age-induced declines in RMR may be attributed to alterations of organ and tissue masses and diminished fat free mass (FFM) which accounts for the magnitude of resting metabolism [3,34]. Previous studies report decreases in RMR in response to negative energy balance and weight loss, with the observed decrease proportional to the energy deficit [35]. When the decline in RMR exceeds the magnitude predicted by the loss of body mass, metabolic adaptation occurs [36,37]. Metabolic adaptation can persist long term, resulting in implications for weight loss [38]. Furthermore, variation in physiological parameters such as: thyroid hormones, growth hormone, serum testosterone, leptin levels and sympathetic nervous system activity contribute to metabolic rate [39,40]. Given the various factors that influence RMR an accurate assessment is important for optimal dietary intake, with particular consideration to be given to the ageing process and associated disease states. Energy imbalance results in weight loss or weight gain and thus a major challenge in helping individuals reduce weight is to help individuals manage their energy balance. The main requirement of a dietary approach to weight reduction is that total energy intake must be less than energy expenditure [39]. Due to the decline in RMR with age (as mentioned above), it may be more important in this age group to measure RMR. Therefore, the aim of this study was to compare the efficacy of a dietary intervention (mRMR versus eRMR) on weight outcomes in ageing Irish adults (50 years and over) with overweight and obesity.

## 2. Materials and Methods

### 2.1. Study Design

This was a single-centre (GMIT) prospective study performed in a population of Irish adults classified as overweight or with obesity. Participants were randomly assigned to a 12-week dietary intervention, where energy intake was established using either (1) mRMR or (2) eRMR. Meal plans with prescribed energy intake and food options in line with habitual patterns were provided to each participant. The protocol consisted of six visits to the clinic which included a screening and familiarisation visit, a nutrition education visit and four measurement visits. Measurement visits were conducted at baseline, week 3, week 6 and week 12 of the dietary intervention. Anthropometric and RMR data were collected across all time points. At baseline and week 12 visits, participants provided capillary blood samples and had their blood pressure measured. The International Physical Activity Questionnaire Short Form (IPAQ-SF) was used to assess physical activity levels at baseline [41]. A 3-day food diary was used to determine habitual energy intake, eating patterns and food preferences at baseline and week 12. Diaries were assessed using Nutritics (Dublin, Ireland) professional dietary analysis software [42]. All measurements were conducted between 8:00 AM and 12:00 AM following an overnight fast and in a voided state. Participants were instructed to refrain from alcohol, nicotine and caffeine, and to avoid strenuous physical activity 10–12 h prior to the measurement visit. This study was conducted in accordance with the ethical principles expressed in the Declaration of Helsinki. Ethics approval was granted by the Research Ethics Committee of Galway Mayo Institute of Technology (GMIT), Ireland (RSC_AC230119). Written informed consent was provided by all participants prior to their inclusion in this study.

### 2.2. Sample Size Calculation

Power analyses were performed prior to the start of this study in order to identify an appropriate sample size. Based on data reported in the literature, 23 individuals were required per group (46 in total) to detect a 5% loss in body weight at a significance level of 0.05 and power of 80%. To allow for a participant withdrawal rate of 20%, the recruitment target was set at 56 participants.

### 2.3. Participants

Fifty-six adults (male *n* = 26; female *n* = 30) were recruited via advertisements in local community centres, libraries, general practitioners and health care centres to take part in this study. Inclusion criteria were adults aged 50 years or greater with a BMI greater than or equal to 25 kg/m^2^. Participants were excluded from this study if they had any health conditions or were taking medication known to influence the measurement of RMR or body composition, experienced weight loss of 5% or greater in the previous 3 months, a past or present history of eating disorders or disorders that would be incompatible with safe and successful participation in this study, as determined by the investigators.

### 2.4. Randomisation and Allocation

Participants were allocated by computer-generated randomisation to one of two intervention groups (1) mRMR or (2) eRMR. The group assignment was stratified using a computerised program (Excel) to ensure equal distribution of BMI in the two groups. The random allocation of the intervention groups was carried out by a separate investigator who was not involved with data collection (LR).

### 2.5. Treatment Protocol

Participants in the mRMR group received a meal plan with energy prescription based on mRMR using a portable IC (ECAL Energy Testing Solutions, UK). The ECAL is a validated open-circuit portable calorimeter that provides practitioners and users with information concerning energy metabolism such as resting energy expenditure and RER. The device utilises breath-by-breath measurement of gas exchange through a plastic mouthpiece and tubing for gas collection. The VO_2_ and VCO_2_ are measured using a small mixing chamber. VO_2_ is measured using a galvanic fuel cell oxygen analyser. VCO_2_ is measured using a patented ultra-low power VCO_2_ analyser which uses Light Emitting Diode (LED) and detector technology in a novel non-dispersive near-infrared absorption sensor. The meal plan consisted of a 7-day menu. Individual energy requirements were calculated from mRMR and a physical activity factor corresponding to a category (low = 1.2, moderate = 1.55, high = 1.725) was applied to participants RMR to account for individual activity requirements. A subsequent energy deficit of 500 kcal was prescribed to promote a 0.45 kg per week weight reduction (mRMR × PAL − 500). Energy requirements were adjusted accordingly following repeated measures of RMR and participants received meal plans to reflect changes required in energy needs. Energy information such as RER was used to advise on dietary modification. Participants with a RER ≥ 0.75 (less than 16% fat burning efficiency) were advised to modify carbohydrate intake. Participants with optimal fat oxidation of >80% were advised to continue to follow healthy eating guidelines while maintaining prescribed energy intake.

Participants in the eRMR group received a meal plan with energy prescription based on eRMR using the Mifflin et al. [28] prediction equation. The meal plan consisted of a 7-day menu following national healthy eating guidelines [43]. The Mifflin et al. [28] prediction equation (males 10 × weight (kg) + 6.25 × height (cm) − 5 × age (years) + 5; females 10 × weight (kg) + 6.25 × height (cm) − 5 × age (years) − 161) was used to inform energy prescription. The eRMR was multiplied by a physical activity level (PAL) factor and a subsequent 500 kcal was subtracted from daily energy requirements to promote a 0.45 kg per week weight reduction (eRMR × PAL − 500).

All participants attended a registered dietician led nutritional educational presentation (1.5 h) at GMIT. Core topics included healthy eating guidelines, weight management, physical activity and common age-related nutrition issues such as constipation, bloating, irritable bowel symptoms and CVD. Physical activity recommendations were provided as per national guidelines, HSE [44].

### 2.6. Compliance

Compliance to the prescribed dietary intervention was monitored from changes in body weight reviewed at week 3 and week 6. Individual consultations of 30 min were carried out at follow-up visits to encourage compliance. The aim of the consultation was to listen to the participant, identify barriers that may be contributing to challenges faced and guide the participant to potential solutions using behaviour change skills and motivational interviewing techniques. Visual aids such as the Irish Food Pyramid, Eat Well plate, disposable cups and food labels were used to encourage the adoption of the dietary guidelines and to describe portion sizes and food choices to the participants. Further individual support was provided via e-mail as required.

### 2.7. Measures

Body weight and anthropometric measurements were assessed at baseline, week 3, week 6 and week 12. Measurements were taken with participants in a fasted and voided state, wearing light clothing and shoes and socks removed. Height was recorded to the nearest 0.5 cm using a stadiometer (Seca Ltd., Birmingham, UK). Body weight and body composition (percentage fat mass and FFM) were measured using bioelectrical impedance analyser (BIA) Tanita BC-418 MA (Tanita UK Limited, Yiewsley, UK). BMI was calculated as weight in kilograms divided by height in metres squared (kg/m^2^). Waist circumference (WC) and hip circumference (HC) were assessed using steel tape (Lufkin W606PM) according to the International Society for the Advancement of Kinanthropometry (ISAK) standards for anthropometric assessment [45].

Resting blood pressure was assessed at baseline and week 12 using an automated sphygmomanometer (Omron M500 HEM-7321-D, Milton Keynes, UK) with participants in a seated position. Three measurements were taken with the average recorded. Blood samples were collected at baseline and week 12 of the dietary intervention. Blood samples were obtained in a fasted state by standard laboratory techniques (finger-stick procedure). Capillary whole blood was tested for fasting blood glucose (mmol/L) using the Accutrend Blood Glucose Monitor (Roche Diagnostics, Dublin, Ireland). Total cholesterol (TC) (mmol/L), triglycerides (mmol/L), HDL (mmol/L), calculated LDL and TC:HDL were assessed using Cardio-Chek monitor (Roche Diagnostics, Dublin, Ireland).

RMR and RER were measured throughout the study period. Participants were instructed to refrain from alcohol and caffeine, and to avoid strenuous physical activity 10–12 h prior to each measurement visit. Participants arrived between 08:00 and 13:00 h following an overnight fast (10–12 h before testing time). Prior to each test, the ECAL calorimeter was calibrated as per manufacturer’s instructions. Following a rest period of 10 min, participants lay in a semi-reclined, comfortable position in a quiet room and were reminded to stay awake. A mouthpiece and nose clip were employed, and participants were instructed to breathe in and out through the mouthpiece as normal. Measurements were recorded for 10 min. Upon completion of the test, the mouthpiece and nose clip were removed.

### 2.8. Statistical Analysis

Analysis were performed using Statistical Package for the Social Sciences (SPSS) for Windows (version 25.0; IBM Corporation, Armonk, NY, USA). Normality of data and outliers were assessed using Shapiro–Wilk and boxplot, respectively. Homogeneity of variances and covariances were assessed by Levene’s test and Box’s M test, respectively. Independent-samples *t*-tests were used to determine differences between the groups at baseline for normally distributed continuous variables. Mann–Whitney U tests were used to assess differences between the groups at baseline for non-normally distributed variables and/or variables with identified outliers. Parametric variables are presented as the mean ± standard deviation (SD) and non-parametric variables as the median (Md) (interquartile range [IQR]). Two-way mixed analysis of variance (ANOVA) assessed the level of difference between groups and within groups overtime using completer analysis for each respective dependent variable. All post hoc tests were carried out with Bonferroni corrections. When sphericity was violated Greenhouse–Geisser correction was reported. Outliers confirmed as genuine data points were included in the analysis. Where data were not normally distributed, the two-way mixed ANOVA was run regardless and reported, as ANOVAs are considered fairly robust to deviations from normality. The level of significance was accepted at *p* < 0.05.

## 3. Results

### 3.1. Participants

**Figure 1:**
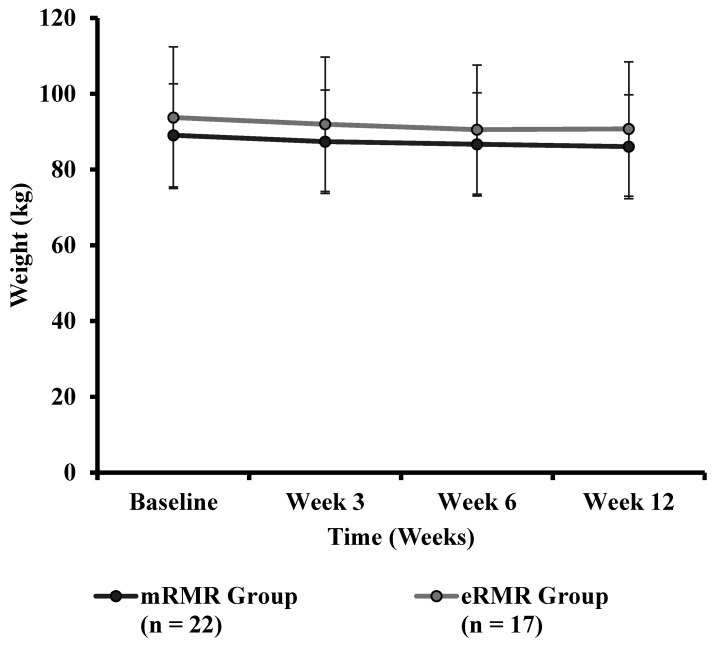
Weight (kg) across the intervention period for the mRMR (*n* = 22) and eRMR (*n* = 17) groups.

**Figure 2:**
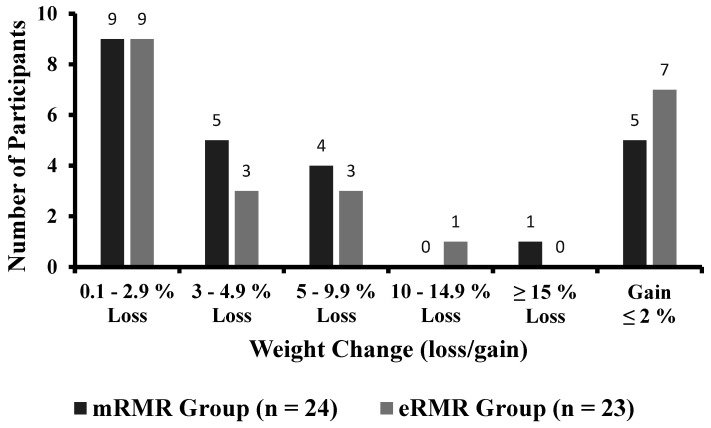
Individual response to weight change for participants completing pre and post measures in the mRMR (*n* = 24) and eRMR groups (*n* = 23).

**Figure 3:**
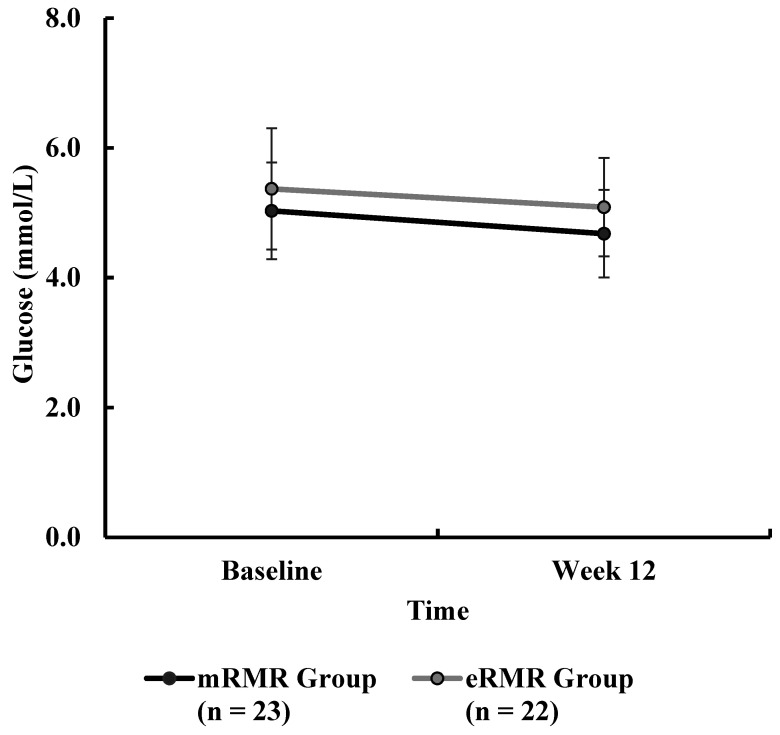
Blood glucose (mmol/L) measured at baseline and week 12 for the mRMR (*n* = 23) and eRMR groups (*n* = 22).

**Figure 4:**
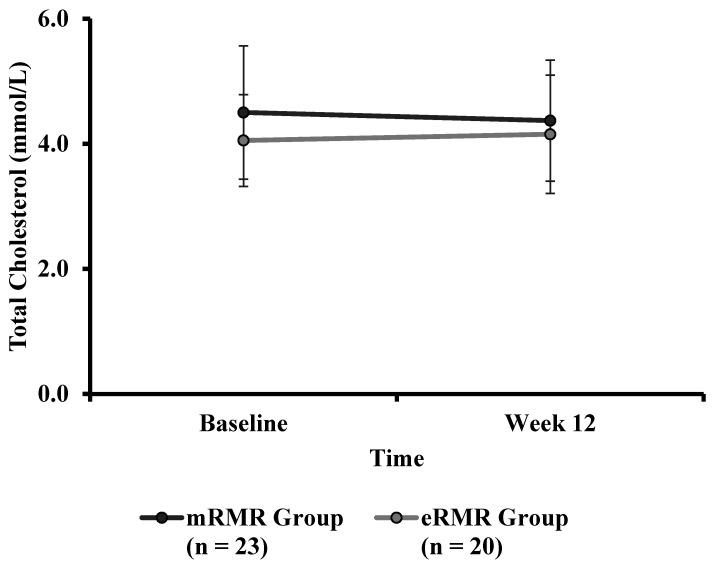
Total cholesterol (mmol/L) measured at baseline and week 12 for the mRMR (*n* = 23) and eRMR groups (*n* = 20).

**Figure 5:**
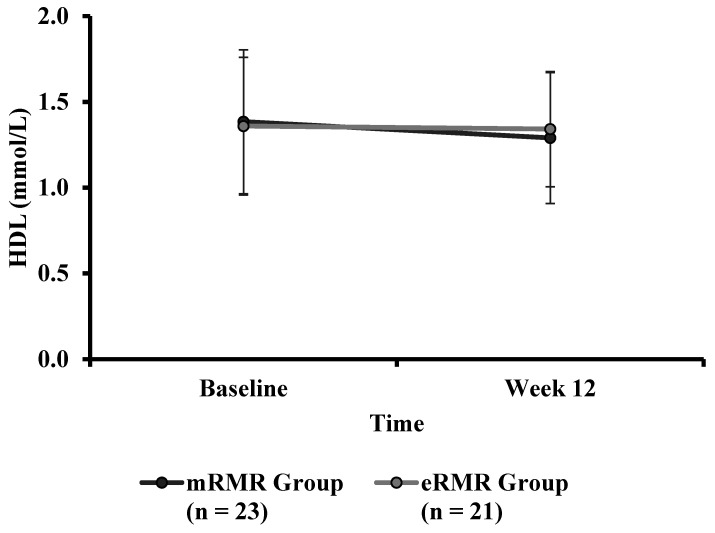
High-density lipoprotein (mmol/L) measured at baseline and week 12 for the mRMR (*n* = 23) and eRMR groups (*n* = 20).

**Figure 6:**
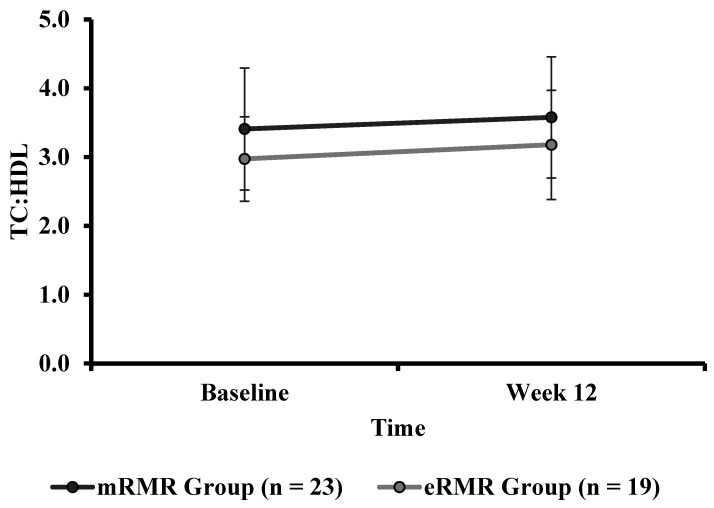
Total cholesterol to high-density lipoprotein ratio calculated at baseline and week 12 in both the mRMR (*n* = 23) and estimated eRMR groups (*n* = 19) groups.

**Figure 7:**
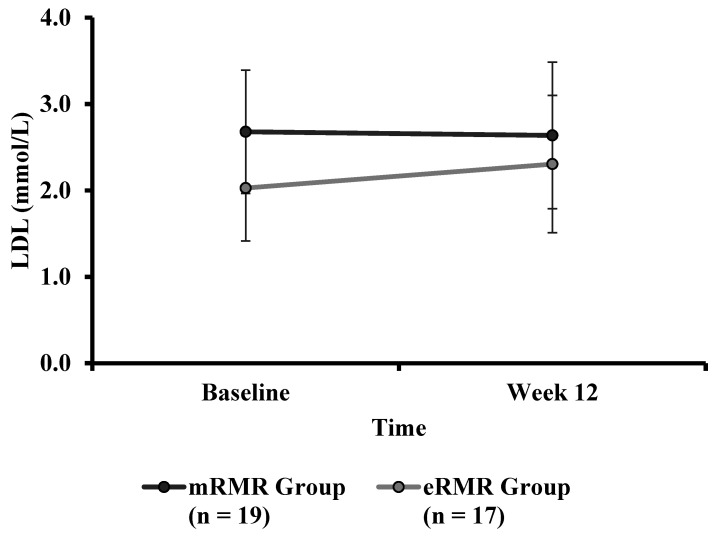
Low-density lipoprotein (mmol/L) calculated at baseline and week 12 for the mRMR (*n* = 19) and eRMR groups (*n* = 17).

**Figure 8:**
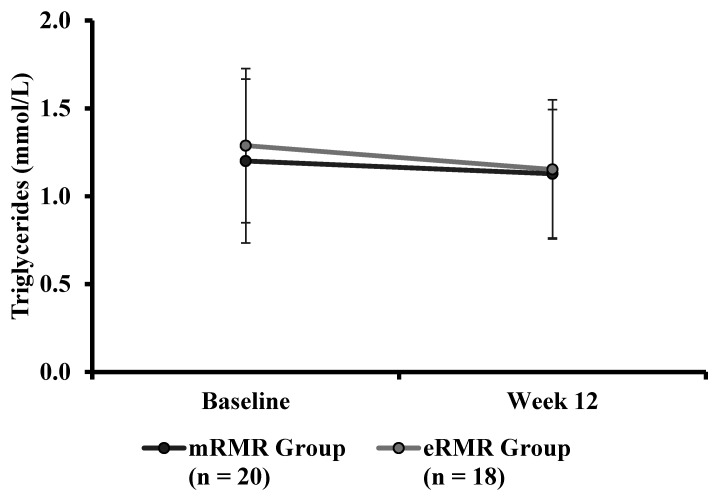
Triglycerides (mmol/L) measured at baseline and week 12 for the mRMR (*n* = 20) and eRMR groups (*n* = 18).

Participant and study flow are illustrated in Supplementary Material Figure S1. From March to April 2019, fifty-six adults were recruited to take part in this study. Two participants (male *n* = 1, female *n* = 1) withdrew prior to baseline testing, citing time commitments conflicting with the study requirements, resulting in fifty-four Caucasian adults (male *n* = 25; female *n* = 29) with a mean ± SD height, body mass, age and BMI of 171.5 ± 9.5 cm, 90.3 ± 15.1 kg, 57.5 ± 6.3 years, and 30.7 ± 4.6 kg/m^2^, respectively, at baseline. Baseline participant characteristics and baseline measures are presented in Table 1. Anthropometric measurements (weight, BMI, WC, HC, WHR, body fat percent and muscle mass) and clinical outcomes (blood pressure) across the intervention period are presented in Table 2. Primary outcome pre and post weight are presented in Table 3. Weight, assessed at baseline, week 3, week 6 and week 12 is illustrated in Figure 1. Individual response to percent weight change is illustrated in Figure 2. Biochemical outcomes at baseline and week 12 are presented in Table 4. Changes in biochemical markers (glucose, TC, HDL, HDL:TC, LDL, triglycerides) are illustrated in Figure 3, Figure 4, Figure 5, Figure 6, Figure 7 and Figure 8, respectively. Metabolic outcomes across the intervention period and estimated energy intake are presented in Table 5. Male and female energy intake at baseline and week 12 and prescribed energy are presented in Table 6. Prescribed energy intake versus reported energy intake from a 3-day food diary is presented in Table 7.

### 3.2. Weight Change

There were no significant differences in baseline weight for mRMR (Md = 88.50, *n* = 29) and eRMR participants (Md = 92.90, *n* = 25), U = 420, z = 0.998, *p* = 0.32, r = 0.14 (Table 2). There was no significant interaction between the intervention groups and time for weight, F(1.478, 54.677) = 0.57, *p* = 0.518, partial η^2^ = 0.02. There was no significant main effect of group on the mean weight F(1, 37) = 0.789, *p* = 0.380, partial η^2^ = 0.021. There was a significant main effect for time on the difference in mean weight at the different time points, F(1.478, 54.677) = 26.726, *p* < 0.0005, partial η^2^ = 0.419. Post hoc analysis revealed that body weight was significantly lower (all < *p* = 0.005) at week 3 (1.9%), week 6 (3.0%) and week 12 (3.3%) compared to baseline for both groups. Weight at week 6 (1.2%) and week 12 (1.4%) was significantly (both < *p* = 0.003) lower than weight at week 3 for both groups. Post hoc analysis revealed no significant difference between weight at week 6 versus week 12 (0.1%, *p* = 1.0) for both groups (Figure 1).

### 3.3. Individual Response to Weight

A total of 37.5% of participants in the mRMR group and 39.1% of participants in the eRMR group reduced between 0.1 and 2.9% of initial body weight. A weight reduction between 3 and 4.9% was observed in 20.8% and 13% of participants in the mRMR and eRMR groups, respectively. A 5–9.9% reduction from initial body weight was observed in 16.7% and 13% of participants in the mRMR and eRMR groups, respectively. Of the participants in the eRMR, 4.3% experienced a 10–14.9% weight reduction. Of the participants in the mRMR 4.2% experienced a weight reduction of ≥15%. Overall, 20.8% mRMR and 17.4% of eRMR participants experienced clinically meaningful (i.e., ≥5% of initial weight) weight reduction. Weight gain of ≤2% was observed in 20.8% and 30.4% of participants in the mRMR and eRMR groups, respectively (Figure 2).

### 3.4. Body Mass Index

There were no significant differences between groups for baseline BMI (Table 1) of mRMR (Md = 106.00, *n* = 28) and eRMR (Md = 112.50, *n* = 25), U = 417, z = 1.194, *p* = 0.232, r = 0.16. There was no significant interaction between the intervention and time on BMI (Table 2), F(1, 45) = 0.335, *p* = 0.566, partial η^2^ = 0.007. There was no significant effect for group, when comparing BMI, F(1, 45) = 0.394, *p* = 0.534, partial η^2^ = 0.009. The main effect of time showed a statistically significant difference in BMI, across time points, F(1, 45) = 25.801, *p* < 0.0005, partial η^2^ = 0.364. Post hoc analysis revealed that BMI was significantly reduced (*p* = 0.001) at week 12 (29.9 kg/m^2^, SE = 0.7) compared to baseline (30.74 kg/m^2^, SE = 7.0).

### 3.5. Waist Circumference

There were no significant differences for WC at baseline (Table 1) between mRMR (Md = 29.30, *n* = 29) and eRMR participants (Md = 29.46, *n* = 25), U = 387, z = 0.43, *p* = 0.67, r = 0.06. There was no significant interaction (intervention x time) for WC (Table 2), F(1, 44) = 1.57, *p* = 0.22, partial η^2^ = 0.03. The main effect of group showed that there was no statistically significant difference in WC between the intervention groups, F(1, 44) = 2.77, *p* = 0.10, partial η^2^ = 0.06. The main effect of time showed a statistically significant difference in WC over time, F(1, 44) = 58.083, *p* < 0.0005, partial η^2^ = 0.569. Post hoc analysis revealed that WC was significantly reduced (*p* = 0.001) at week 12 (−9.3 cm) compared to baseline (111.1 cm SE = 1.9).

### 3.6. Hip Circumference

There was no significant difference in HC at baseline (Table 1) between mRMR (Md = 112.25, *n* = 28) and eRMR participants (Md = 113.00, *n* = 25), U = 351, z = 0.027, *p* = 0.979, r = 0.00. There was no significant interaction between the intervention and time on HC (Table 2), F(1, 44) = 0.010, *p* = 0.921, partial η^2^ = 0.000. The main effect of group showed that there were no statistically significant differences in HC between the intervention groups (Table 2), F(1, 44) = 0.000, *p* = 0.99, partial η^2^ = 0.000. The main effect of time showed a substantial statistically significant difference in HC over time (Table 2), F(1, 44) = 20.586, *p* < 0.0005, partial η^2^ = 0.32. Post hoc analysis revealed that HC was significantly reduced (*p* = 0.001) at week 12 (−8.4 cm) compared to baseline (117.1 cm, SE = 2.1).

### 3.7. Waist to Hip Ratio

There were no significant differences in WHR between mRMR (0.93 ± 0.077) and eRMR groups (0.96 ± 0.082; t(51) = −1.479, *p* = 0.145) at baseline (Table 1). There was no significant interaction between the intervention and time on WHR (Table 2), F(1, 44) = 2.528, *p* = 0.119, partial η^2^ = 0.054. The main effect of group showed a substantial significant difference in WHR between the intervention groups, F(1, 44) = 5.751, *p* = 0.02, partial η^2^ = 0.12. The main effect of time showed a substantial statistically significant difference in WHR at the different time points, F(1, 44) = 4.085, *p* = 0.049, partial η^2^ = 0.09. Post hoc analysis revealed that WHR was significantly reduced (*p* = 0.049) at week 12 (0.9 SE = 0.01) compared to baseline (1.0 SE = 0.01).

### 3.8. Percent Body Fat

There were no significant differences in baseline body fat percent (Table 1) between mRMR group (37.53 ± 8.57) and eRMR groups (35.72 ± 7.53; t(52) = 0.818, *p* = 0.417). There was no significant interaction between the intervention groups and time on body fat (Table 2), F(1.785, 66.044) = 0.705, *p* = 0.482, partial η^2^ = 0.019. The main effect of group showed that there was no significant difference in body fat between the intervention groups, F(1, 37) = 0.084, *p* = 0.773, partial η^2^ = 0.002. The main effect of time showed no significant difference in body fat over time, F(1.785, 66.044) = 2.259, *p* = 0.118, partial η^2^ = 0.058.

### 3.9. Muscle Mass

There were no significant differences in baseline muscle mass (Table 1) between mRMR (Md = 49.10, *n* = 29) and eRMR participants (Md = 59.60, *n* = 25), U = 433, z = 1.22, *p* = 0.221, r = 0.17. There was no significant interaction between the intervention groups and time on muscle mass (Table 2), F(1.980, 73.275) = 1.017, *p* = 0.366, partial η^2^ = 0.027. The main effect of group showed that there was no significant difference in muscle mass between the intervention groups, F(1, 37) = 0.846, *p* = 0.36, partial η^2^ = 0.02. The main effect of time showed a substantial significant difference in muscle mass over time, F(1.980, 73.275) = 6.227, *p* < 0.0005, partial η^2^ = 0.14. Post hoc analysis revealed that muscle mass was significantly reduced (both < *p* = 0.035) at week 3 (0.8 kg) and week 12 (2.1 kg) compared to baseline (55.6 kg, SE = 2.0). Post hoc analysis revealed no significant difference between muscles mass at week 6 and week 12 versus week 3 (both > *p* = 0.65). Post hoc analysis revealed no significant difference between muscle mass at week 6 versus week 12 (0.1%, *p*= 0.21).

### 3.10. Systolic Blood Pressure

There was a significant difference in systolic blood pressure at baseline (Table 1) between mRMR (Md = 126.00, *n* = 29) and eRMR participants (Md = 135.00, *n* = 25), U = 481, z = 2.065, *p* = 0.039, r = 0.28. There was no significant interaction between the intervention and time on systolic blood pressure (Table 2), F(1, 44) = 1.124, *p* = 0.295, partial η^2^ = 0.025. The main effect of group showed a substantial significant difference in systolic blood pressure between the intervention groups, F(1, 44) = 6.654, *p* = 0.013, partial η^2^ = 0.13. Post hoc analysis revealed systolic blood pressure was significantly lower in the mRMR group when compared to the eRMR group (−11.71 mmHg, *p* = 0.01). The main effect of time showed a significant difference in systolic blood pressure over time, F(1, 44) = 6.305, *p* < 0.0005, partial η^2^ = 0.13. Post hoc analysis revealed that systolic blood pressure was significantly reduced at week 12 (−4.9 mmHg, *p* = 0.02) compared to baseline (130 mmHg, SE = 2.7).

### 3.11. Diastolic Blood Pressure

There were no significant differences in baseline diastolic blood pressure (Table 1) between mRMR (Md = 83.00, *n* = 29) and eRMR participants (Md = 87.00, *n* = 25), U = 457, z = 1.65, *p* = 0.099, r = 0.22. There was no significant interaction between the intervention and time on diastolic blood pressure (Table 2), F(1, 44) = 0.022, *p* = 0.884, partial η^2^ = 0.000. The main effect of group showed a substantial statistically significant difference in diastolic blood pressure between the intervention groups, F(1, 44) = 4.524, *p* = 0.039, partial η^2^ = 0.093. Post hoc analysis revealed that diastolic blood pressure was significantly lower in the eRMR group when compared to mRMR (−5.7 mmHg, *p* = 0.04). The main effect of time showed a significant difference in diastolic blood pressure over time, F(1, 44) = 5.444, *p* < 0.0005, partial η^2^ = 0.110. Post hoc analysis revealed diastolic blood pressure was significantly reduced at week 12 (−3.1 mmHg, *p* = 0.02) compared to baseline (83.96 mmHg, SE 1.6).

### 3.12. Blood Glucose

There were no significant differences in baseline blood glucose (Table 1) between mRMR (Md = 4.85, *n* = 28) and eRMR participants (Md = 5.30, *n* = 25), U = 392, z = 0.749, *p* = 0.454, r = 0.10. There was no statistically significant interaction between the intervention and time on blood glucose concentration (Table 4), F(1, 43) = 0.112, *p* = 0.739, partial η^2^ = 0.003. The main effect of group showed no significant difference in blood glucose concentration between the intervention groups, F(1, 43) = 3.203, *p* = 0.081, partial η^2^ = 0.069. The main effect of time showed a statistically significant difference in blood glucose concentration over time, F(1, 43) = 9.120, *p* < 0.0005, partial η^2^ = 0.175. Post hoc analysis revealed that blood glucose was significantly lower at week 12 (−0.3 mmol/L, *p* = 0.004) compared to baseline (5.2 mmol/L, SE 0.12) (Figure 3).

### 3.13. Total Cholesterol

There was a significant difference in baseline total cholesterol between mRMR (Table 1) (Md = 4.74, *n* = 28) and eRMR participants (Md = 3.935, *n* = 22), U = 186, z = −2.385, *p* = 0.017, r = 0.34. There was no significant interaction between the intervention and time on total cholesterol concentration (Table 4), F(1, 41) = 0.584, *p* = 0.449, partial η^2^ = 0.014. The main effect of group showed no significant difference in total cholesterol between the intervention groups, F(1, 41) = 1.836, *p* = 0.183, partial η^2^ = 0.043. The main effect of time showed no significant difference in total cholesterol over time, F(1, 41) = 0.011, *p* = 0.918, partial η^2^ = 0.000 (Figure 4).

### 3.14. High-Density Lipoprotein

There were no significant differences in baseline HDL (Table 1) between mRMR (Md = 1.34, *n* = 28) and eRMR participants (Md = 1.23, *n* = 23), U = 306, z = −0.303, *p* = 0.762, r = −0.04. There was no statistically significant interaction between the intervention and time on HDL (Table 4), F(1, 42) = 2.196, *p* = 0.146, partial η^2^ = 0.050. The main effect of group showed that there was no significant difference in HDL (Figure 5) between the intervention groups F(1, 42) = 0.014, *p* = 0.908, partial η^2^ = 0.000. The main effect of time showed a significant difference in HDL concentration over time, F(1, 42) = 4.659, *p* < 0.0005, partial η^2^ = 0.100. Post hoc analysis revealed that HDL was significantly lower at week 12 (−0.1 mmol/L, *p* = 0.04) compared to baseline (1.4 mmol/L, SE 0.1).

### 3.15. Total Cholesterol to High-Density Lipoprotein Ratio

There were no significant differences in baseline TC:HDL (Table 1) between mRMR (Md = 3.4, *n* = 28) and eRMR participants (Md = 3.0, *n* = 21), U = 206, z = −1.771, *p* = 0.077, r = 0.25. There was no statistically significant interaction between the intervention and time on TC:HDL (Table 4), F(1, 40) = 0.028, *p* = 0.869, partial η^2^ = 0.001. The main effect of group showed no significant difference in TC:HDL between the intervention groups, F(1, 40) = 3.383, *p* = 0.073, partial η^2^ = 0.078. The main effect of time showed no significant difference in TC:HDL over time (Table 2), F(1, 40) = 3.043, *p* = 0.089, partial η^2^ = 0.071 (Figure 6).

### 3.16. Low-Density Lipoprotein

There was a significant difference in baseline LDL (Table 1) between mRMR (Md = 2.77, *n* = 24) and eRMR participants (Md = 2.21, *n* = 19), U = 104, z = −3.033, *p* = 0.002, r = 0.50. There was no statistically significant interaction between the intervention and time on LDL (Table 4), F(1, 34) = 1.194, *p* = 0.282, partial η^2^ = 0.034. The main effect of group showed that there was a substantial statistically significant difference LDL between the intervention groups, F(1, 34) = 5.864, *p* = 0.021, partial η^2^ = 0.15. Post hoc analysis revealed that LDL was significantly lower in the eRMR group than the mRMR group (–.491 mmol/L, *p* = 0.021). The main effect of time showed no statistically significant difference in LDL over time, F(1, 34) = 0.642, *p* = 0.429, partial η^2^ = 0.019 (Figure 7).

### 3.17. Triglycerides

There were no significant differences in baseline triglycerides (Table 1) between mRMR (Md = 1.08, *n* = 25) and eRMR participants (Md = 1.19, *n* = 19), U = 278, z = 0.960, *p* = 0.337, r = 0.14. There was no significant interaction between the intervention and time on triglyceride concentration, F(1, 36) = 0.186, *p* = 0.669, partial η^2^ = 0.005 (Table 4). The main effect of group showed no significant difference in triglyceride concentration between the intervention groups F(1, 36) = 0.242, *p* = 0.626, partial η^2^ = 0.007. The main effect of time showed no significant difference in triglyceride concentration over time, F(1, 36) = 2.028, *p* = 0.163, partial η^2^ = 0.053, (Figure 8).

### 3.18. Energy Intake

There were no significant differences in baseline energy intake (Table 1) of mRMR (Md = 2195.0, *n* = 21) and eRMR (Md = 2129.0, *n* = 19), U = 184, z = −0.420, *p* = 0.68, r = 0.07. There was no significant interaction between the intervention and time on energy intake (Table 5), F(1, 28) = 0.003, *p* = 0.956, partial η^2^ = 0.000. The main effect of group showed no significant difference in energy intake between the intervention groups, F(1, 28) = 1.225, *p* = 0.279, partial η^2^ = 0.042. The main effect of time showed a substantial statistically significant difference in energy intake at the different time points, F(1, 28) = 14.934, *p* < 0.0005, partial η^2^ = 0.348. Post hoc analysis revealed that energy intake was significantly lower at week 12 compared to baseline (−479 kcal/day, *p* = 0.001).

### 3.19. Measured Resting Metabolic Rate

There were no significant differences in baseline measured RMR (Table 1) between mRMR (Md = 1604.00, *n* = 29) and eRMR participants (Md = 1691.00, *n* = 23), U = 347, z = 0.249, *p* = 0.804, r = 0.04. There was no significant interaction between the intervention groups and time on measured RMR (Table 5), F(2.421, 87.171) = 0.284, *p* = 0.794, partial η^2^ = 0.008. The main effect of group showed that there was no significant difference for measured RMR between the intervention groups, F(1, 36) = 0.101, *p* = 0.752, partial η^2^ = 0.003. The main effect of time showed no significant difference for measured RMR over time, F(2.421, 87.171) = 1.525, *p* = 0.220, partial η^2^ = 0.041.

### 3.20. Respiratory Exchange Ratio

There were no significant differences in baseline measured RER (Table 1) between mRMR (Md = 0.77, *n* = 29) and eRMR participants (Md = 0.81, *n* = 23), U = 381, z = 0.876, *p* = 0.381, r = 0.12. There was no significant interaction between the intervention groups and time on RER (Table 5), F(2.339, 84.206) = 1.112, *p* = 0.340, partial η^2^ = 0.030. The main effect of group showed that there was no significant difference in measured RER between the intervention groups, F(1, 36) = 0.105, *p* = 0.748, partial η^2^ = 0.003. The main effect of time showed no significant difference in RER over time, F(2.339, 84.206) = 1.159, *p* = 0.324, partial η^2^ = 0.031.

### 3.21. Predicted Resting Metabolic Rate

There were no significant differences in RMR predicted by Mifflin et al. (1990) at baseline (Table 1) between mRMR group (1560.28 ± 221.71) and eRMR groups (1639.25 ± 272.21; t(51) = −1.164, *p* = 0.250, mean difference = −78.97 (95% CI, −215.13 to 57.18). There was no significant interaction between the intervention and time on eRMR (Table 5), F(1.83, 67.60) = 0.215, *p* = 0.787, partial η^2^ = 0.837. The main effect of group showed that there was no statistically significant difference in eRMR between the intervention groups, F(1, 37) = 0.841, *p* = 0.365, partial η^2^ = 0.022. The main effect of time showed a significant difference in eRMR over time, F(1.83, 84) = 12.88, *p* < 0.0005, partial η^2^ = 0.258. Post hoc analysis revealed that eRMR was significantly lower (both < *p* = 0.0005) at week 6 (1.8%), and week 12 (1.9%) compared to baseline. Post hoc analysis revealed no significant difference between eRMR at week 3 versus baseline (0.3%, *p*= 1.0). Post hoc analysis revealed that eRMR at week 6 (1.4%) and week 12 (1.6%) was significantly (both < *p* = 0.023) lower than eRMR at week 3. Post hoc analysis revealed no significant difference between eRMR at week 6 versus week 12 (0.2%, *p* = 1.0).

## 4. Discussion

The aim of this study was to compare the efficacy of a dietary intervention (mRMR versus eRMR) on weight outcomes in Irish adults aged 50 years and over with overweight and obesity. The primary outcome of this study indicates that employing a reduced-calorie diet using IC to determine energy needs when improving weight outcomes in adults with overweight and obesity is equal to employing a reduced-calorie diet based on the Mifflin et al. [28] prediction equation. Following the study period, a significant (*p* < 0.05) reduction in body weight was observed in both mRMR (−4.2% of initial body weight) and eRMR (−3.2%) groups. However, there were no significant (*p* ≥ 0.05) differences between groups. Overall, 20.8% and 17.4% of mRMR and eRMR participants, respectively, experienced clinically meaningful weight reduction. One participant in the eRMR group experienced a 10–14.9% weight reduction, and one participant in the mRMR group experienced a more than 15% weight reduction. Rapid weight loss may be a sign of underlying health conditions or chronic disease. No health condition, disease or illness was identified prior to or during the intervention that may be attributed to rapid or unintentional weight loss. From the one-to-one consultations, it can be assumed that the observed weight loss may be attributed to successful adherence to diet and lifestyle modifications. While a secondary analysis of data assessing biological sex differences in weight variation within the mRMR and eRMR groups was not investigated in the present study, a previous study [46] with similar participants investigated gender differences in weight and BMI variation in response to a dietary intervention based on measured RMR using IC and equations. No statistically significant differences in body weight and BMI variation between the two IC and no IC groups were found between males and females (three-way interaction time by treatment by gender: *p* = 0.16 for BMI, *p* = 0.11 for weight), although a trend to a greater weight loss in females was observed in both groups. Secondary outcome measures revealed a significant reduction (*p* ≤ 0.05) in BMI, WC and muscle mass in both groups. Differences observed between groups were not significant (*p* ≥ 0.05). There were no significant (*p* ≥ 0.05) differences in percent body fat over time or between groups. Both groups experienced a significant (*p* ≤ 0.05) reduction in systolic and diastolic BP following the intervention period. Blood glucose and triglycerides were significantly (*p* ≤ 0.05) lower in both groups and there was no significant (*p* ≥ 0.05) difference observed between groups. There was no significant (*p* ≥ 0.05) difference for total cholesterol or TC:HDL over time or between groups. HDL concentration was significantly (*p* < 0.05) lower in both groups with no significant difference (*p* ≥ 0.05) between groups. No significant difference (*p* ≥ 0.05) was observed for LDL over the study period. However, there were significant (*p* < 0.05) differences in LDL between groups at baseline, and 47% of participants in the eRMR group displayed an upward trend in LDL over the study period (Figure 3).

In contrast, previous studies reported significant between-group differences when comparing similar dietary interventions (i.e., prediction equations versus metabolic based) in a comparable population [46,47]. Participants following a nutrition plan based on eRMR for a period of 90 days experienced a 2% weight reduction compared to −4.5% when following a diet based on mRMR [47]. The between-group differences may be explained by the methodical differences used to estimate RMR. Massarini et al. [46] employed the Harris and Benedict [48] prediction equation while McDoniel et al. [47] employed the American College of Chest Physicians (ACCP) prediction equation (25 × baseline body weight (kg) − 250 to 500 kcal/day) [5,49]. The application of prediction equations provides a source of variability as often they are utilised for a population which they were not originally developed for, thus resulting in reduced accuracy among specific populations [50,51]. Later work conducted by McDoniel et al. [52] in a similar population reported similar results to the present study. McDoniel et al. [52] conducted a 24 week randomised controlled trial, where usual care participants received a fixed low-calorie diet (i.e., 1200 kcal/day for females and 1600 kcal/day for males, respectively) and participants in the metabolic diet (MD) group received an individualised nutrition plan based on mRMR. McDoniel and Hammond [52] reported a significant reduction in body weight at week 12, but similar to the current study observed no significant differences between the intervention groups. When comparing usual care practice to eRMR, participants following the fixed-calorie diet experienced a greater weight reduction (1.3%). Participants in the usual care group experienced a 4.5% reduction in bodyweight compared to the eRMR group (−3.2%) in the present study. It is reasonable to assume that the greater weight reduction observed by McDoniel and Hammond [52] in the usual care group may be attributed to a greater energy deficit compared to participants in the eRMR group. Usual care participants were prescribed approximately 300–500 kcal/day less than eRMR participants (Table 7) (females: 1500 ± 141 kcal/day; males: 2100 ± 236 kcal/day). Standardised hypocaloric balanced diets are designed to facilitate a 0.5–1.0 kg per week weight reduction by consuming approximately 500 kcal/day less than required for weight maintenance. This is based on the assumption that a negative energy balance of 7700 kcal is required for a 1 kg reduction in body weight. Therefore, an energy deficit of 3500 kcal/week should result in a 0.5 kg/week weight reduction. McDoniel et al. [53] prescribed similar energy intakes to that described in this study and observed comparable results. Participants in the self-monitoring and RMR technology (SMART) group received a nutrition plan based on mRMR and usual care participants were prescribed a standardised ad libitum diet (females:1200 kcal/day; males:1600 kcal/day). Participants in the SMART group experienced a 3.9% weight reduction, slightly lower than but similar to participants in the present study (−4.2%) employing comparable metabolic based diets (mRMR). Energy prescription was based on RMR measured by IC, which may explain the similarities. For instance, women in the mRMR and SMART group were prescribed 1584 ± 271 kcal/day and 1656 ± 334 kcal/day, respectively, while men in the mRMR and SMART group were prescribed 2180 ± 253 kcal/day and 2296 ± 565 kcal/day, respectively. The current study observed a weight gain equal to or less than 2% of initial body weight in 20.8% and 30.4% of participants in the mRMR and eRMR groups, respectively. Factors possibly contributing to an increase in body weight despite a reduced energy prescription include difficulty in adopting positive behaviour change to support dietary changes and thus influence weight.

Secondary outcomes of this study support previous research demonstrating that modest weight reduction lowers blood pressure, triglycerides and glucose [54]. A reduction in systolic (mRMR −2.8 ± 1.1 mmHg; eRMR: −7.0 ± 4.3 mmHg) and diastolic (mRMR: −3.3 ± 2.6; eRMR: 2.9 ± 0.0 mmHg) blood pressure, triglycerides and glucose was observed (Table 2 and Table 4). These outcomes may be attributed to components of the dietary intervention which emphasise high fruit and vegetable consumption and intake of wholegrains, while reduced sodium and saturated fat intake, and limited intake of energy dense foods. A high intake of vegetables and fruit is associated with reduced blood pressure and a lower risk of CVD. Furthermore, dietary fibre intake and consumption of wholegrain products are linked to a lower risk of diabetes and reduced diastolic blood pressure, while lowering sodium intake reduces blood pressure [55]. Similar to Zinn et al. [56] a non-significant upward and downward trend in LDL was observed in the eRMR and mRMR groups, respectively, with 47% of participants in the eRMR group displaying an upward trend of LDL and 31.6% of participants in the mRMR group showing a downward trend in LDL (Figure 8). A systematic literature review evaluating the effect of energy restriction diets on weight loss outcomes in adults with overweight and obesity reported significant reductions in FFM or lean body mass in six of the included studies (*n* = 216) [57]. Muscle mass was significantly reduced at week 3 (0.8 kg) and week 12 (2.1 kg) compared to baseline (Table 2). Loss of FFM is unfavourable for numerous reasons including the impact on metabolic health, functional capacity, i.e., the ability to carry out activities of daily living and the increased risk of injury associated with reduced functional capacity. Greater FFM is linked to a higher metabolic rate, which is advantageous for weight reduction. In an effort to offset the potential loss of FFM, the present study encouraged adherence to current national physical activity guidelines. National physical activity guidelines for older adults recommend at least 30 min a day of moderate-intensity activity on five days a week, or 150 min per week. The addition of moderate-intensity aerobic exercise, primarily walking, to intentional weight loss has been shown to attenuate the loss of muscle mass in older adults with overweight and obesity [58]. Aerobic activity, muscle-strengthening and balance activities form part of the recommendations for adults (Get Ireland Active, HSE). Despite the effort made by participants in this study to attenuate the effects of lean mass loss, a significant decrease was observed.

There was no significant difference in RMR measured by IC at baseline or across the intervention period for both groups (Table 1 and Table 5). This observation supports previous studies that suggest resting energy expenditure remains relatively stable following modest energy deficit diet. Severe energy restriction is associated with greater loss in metabolically active tissue, and thus results in metabolic adaptation [59]. A recent systematic review demonstrated that resistance exercise was effective for increasing RMR. However, the same was not apparent for aerobic exercise [60]. This is an important finding given that the addition of physical activity to weight management programmes is an accepted practice to encourage energy expenditure. It is unclear whether the addition of resistance exercise specifically to weight reduction programmes may attenuate metabolic adaptation by increasing RMR. Future research is required to determine the extent to which resistance exercise affects metabolic adaptation of RMR related to energy restriction.

RMR estimated using the Mifflin et al. [28] prediction equation was lower than actual RMR for both groups (Table 5). eRMR was significantly lower at weeks 6 and 12 compared to baseline for participants in both the mRMR and eRMR groups. If eRMR was lower than actual mRMR, then participants following a diet based on estimated energy needs would receive a dietary plan underestimating calorie requirement and, therefore, greater weight loss would be anticipated. When separated out, male and female analysis of energy intake estimated using 3-day food diaries indicate that both male and female eRMR participants were under their prescribed intake at week 12, leading to further deficit than required (Table 7). Given that similar weight loss was evident between the eRMR and mRMR groups, this would suggest that the same weight loss may occur with a higher-calorie plan.

Furthermore, results from this study suggest energy intake closely matched to mRMR results in higher level of dietary adherence in male participants. Estimated male energy intake (2052 ± 597 kcal) from 3-day food diaries was very similar to energy intake prescribed using mRMR which is advantageous in weight reduction strategies. Strong adherence level is associated with successful outcomes in weight management [61]. A recent study investigating sociocultural gender factors which influence food behaviours, such as dietary preferences and adherence, reported that men were more adherent to a healthy low-carbohydrate (HLC) than women (*p* = 0.02) vs. healthy low-fat (HLF) diet [62]. Another possible reason for this may be the addition of regular one-to-one support provided by the researcher, where goal setting and self-monitoring of food intake were encouraged using motivational interviewing techniques and behavioural change skills. This may not be the case for women as estimated energy intake for females (1736 ± 473 kcal) was higher than prescribed and indicates that additional support may be warranted to aid adherence to weight management programmes for females. Assessment of energy intake is often unreliable, particularly in individuals classified as overweight or affected by obesity [14]. Therefore, a greater frequency of dietary self-monitoring, which is associated with greater weight loss success, may benefit females [63].

### 4.1. Strengths of Study

This research was unique in that it was the first study to compare RMR estimated by the Mifflin et al. (1990) prediction equation using actual body weight to inform dietary prescription directly to dietary prescription based on measured RMR value in this specific population group, namely Irish adults aged 50 years and over with a BMI ≥ 25 kg/m^2^ and of Caucasian ethnicity. Thus, the results of this study may be extrapolated to Irish adults aged 50 years or over with a BMI greater than 25 kg/m^2^. A major strength of this research was the level of adherence to the intervention, as a 93% retention rate was observed. For power based on weight reduction calculations and allowing for a 20% drop out, 46 people were required in total (approximately 23 in each intervention group). Fifty-two participants completed this study, thus allowing for statistical significance. Extensive support was provided by the researcher to all participants during this study, with regular one-to-one support, individualised nutrition coaching and accessible to participants should they have any questions or concerns. The researcher was certified in motivational interviewing and behaviour change skills, which, alongside a non-judgemental patient-centred approach, enhanced the consultation process and gave the participants an opportunity to engage in the conversation about health concerns relating to their weight. McGowan [64] highlighted the importance of strong communication skills, avoiding stigmatisation and the appropriate use of person-first language as imperative to successfully engaging patients. This level of engagement allowed for individual adjustments to be made where necessary and highlights that, irrespective of how the calorie deficit is achieved, it is the individualised and tailored approach that is the most important factor for retention and thus achieving a successful outcome. Further strengths of this study include consistent data collection by the same researcher, which reduces potential measurement error.

### 4.2. Limitations

There are a few limitations to this study. A secondary analysis of data for biological sex differences in weight variation within the mRMR and eRMR groups, or other sex-related factors, such as genotype, hormones, metabolic syndrome, or psychosocial factors that may affect either adherence to dietary intervention or weight reduction response were not investigated. Aronica et al. [62] highlighted the need for such analysis while acknowledging that a limited amount of weight reduction studies demonstrated sufficient power to compare the effects of energy restriction diets on weight outcomes in women vs. men. The present study duration was 12 weeks, thus it is unknown whether participants in the mRMR or eRMR groups maintained weight reduction over a longer period. While qualitative measures were collected at baseline to estimate the energy expenditure associated with physical activity and to inform the subsequent dietary intervention accurately, measuring energy expenditure associated with physical activity throughout this study using measurement devices such as the gold-standard doubly labelled water method or wearable monitors such as accelerometers and movement sensors was beyond the scope of this study. Some participants may have become more physically activity as this study progressed and so the contributory mechanism of physical activity energy expenditure to weight outcome remains speculative. Finally, all participants in this study were of a specific age range (≥50 years), BMI class (25 kg/m^2^) and ethnic group (Caucasian) and thus caution should be used when extrapolating these results to other population groups.

### 4.3. Future Direction

Based on these data, the use of RMR technology demonstrates promise for effective weight reduction outcomes. Future research is needed to better understand the efficacy of RER and substrate utilisation information in tailoring dietary prescription. To address the gap in the literature as identified by Aronica et al. [62] biological sex differences such as body composition and metabolism should be investigated among research participants to compare the effects of diets with energy prescription based on IC or prediction equations on weight outcomes. Furthermore, sociocultural gender factors which influence food behaviours such as dietary preferences and adherence should be explored. The high retention rate (93%) demonstrated in this study would suggest that participants are more likely to adhere to a modest calorie-deficit nutrition programme with regular support. The effects over longer time frames are less clear and future research is required to investigate whether compliance to a modest calorie deficit is more suited to older adult population long term. Future research should accurately measure physical activity energy expenditure to determine its contribution to weight outcomes. Additional research is needed to determine the extent to which resistance exercise affects metabolic adaptation of RMR related to energy restriction.

## 5. Conclusions

In conclusion, the results of this study suggest that a reduced-energy diet based on mRMR or eRMR facilitates weight reduction in adults aged ≥ 50 years over the short term (12 weeks) and favours a reduction in blood pressure, triglycerides and glucose, thus reducing CVD risk factors. Overall, 20.8% mRMR and 17.4% of eRMR participants experienced clinically meaningful (i.e., ≥5% of initial weight) weight reduction. Moreover, dietary approaches that entail modest calorie deficit combined with individual counselling using MI and behaviour change skills that support and encourage small behaviour changes may be effective in short-term (up to 12 weeks) adult (aged ≥ 50 years) weight management.

## Figures and Tables

**Table 1:** Baseline participant characteristics.

	*n*	mRMR Group	*n*	eRMR Group	*p* Value
Sex males (M) females (F)	M10/F19		M15/F10		
Age (years)	29	56.7 ± 5.3	25	58.6 ± 7.1	
Height (cm)	29	170.3 ± 9.5	25	173.1 ± 8.8	
Weight (kg)	29	88.5 (81.0, 94.8)	25	92.9 (81.0, 98.9)	† *p* = 0.32
BMI (kg/m^2^)	29	29.3 (26.8, 33.4)	25	29.5 (27.7, 32.6)	† *p* = 0.23
WC (cm)	28	106.0 (99.3, 115.3)	25	112.5 (102.5, 116.0)	† *p* = 0.67
HC (cm)	28	112.3 (106.0, 126.1)	25	113.0 (107.3, 123.0)	† *p* = 0.98
WHR	28	0.9 ± 0.1	25	1.0 ± 0.1	ᶲ *p* = 0.15
Body Fat (%)	29	37.5 ± 8.6	25	35.7 ± 7.5	† *p* = 0.42
Muscle Mass (kg)	29	49.1 (45.1, 63.0)	25	59.6 (44.6, 67.3)	ᶲ *p* = 0.22
BPsys (mmHg)	29	126.0 (115.5, 136.0)	25	135.0 (124.0, 151.0)	† *p* = 0.04
BPdia (mmHg)	29	83.0 (74.5, 87.5)	25	87.0 (78.0, 92.0)	† *p* = 0.10,
Glucose (mmol/L)	28	4.9 (4.5, 5.5)	25	5.3 (4.5, 5.8)	† *p* = 0.45
TC (mmol/L)	28	4.7 (4.1, 5.3)	22	3.9 (3.5, 4.5)	† *p* = 0.02
HDL (mmol/L)	28	1.3 (1.1, 1.8)	23	1.2 (1.1, 1.6)	† *p* = 0.76
TC:HDL Ratio	28	3.4 (2.6, 4.1)	21	3.0 (2.4, 3.4)	† *p* = 0.08
LDL (mmol/L)	24	2.8 (2.5, 3.1)	19	2.2 (1.7, 2.6)	† *p* = 0.00
TG (mmol/L)	25	1.1 (0.9, 1.4)	19	1.2 (1.0, 1.6)	† *p* = 0.34
mRMR (kcal)	29	1604.0 (1374.0, 2011.5)	23	1691.0 (1455.0, 2067.0)	† *p* = 0.80
RER	29	0.8 (0.7, 0.9)	23	0.8 (0.7, 0.9)	† *p* = 0.38
eRMR kcal	29	1560.3 ± 221.7	24	1639.3 ± 272.2	ᶲ *p* = 0.25
Energy Intake (kcal)	21	2195.0 (1863.5, 2755.0)	19	2129.0 (1880.0, 2586.0)	† *p* = 0.68

Values are presented as the mean ± SD or median (25th–75th percentile), if data were non-parametric. *n* = number of participants with data available for each outcome. IQR, interquartile range; BMI, body mass index; WC, waist circumference; HC, hip circumference; WHR, waist to hip ratio; BPsys, systolic blood pressure; BPdia, diastolic blood pressure; TC, total cholesterol; HDL, high-density lipoprotein; TC:HDL, total cholesterol to high-density lipoprotein ratio; LDL, low-density lipoprotein; TG, triglycerides; RMR, resting metabolic rate; RER, respiratory exchange ratio; ᶲ denotes independent-samples *t*-test; † denotes Mann–Whitney U test.

**Table 2:** Anthropometric and clinical outcomes across the intervention period.

	mRMR Group					eRMR Group							
	*n*	Baseline	Week 3	Week 6	Week 12	*n*	Baseline	Week 3	Week 6	Week 12	Time Effect, *p*	Group Effect, *p*	Time-by-Group Interaction, *p*
Weight (kg)	22	89.0 ± 13.6	87.3 ± 13.7	86.7 ± 13.6	86.0 ± 13.7	17	93.7 ± 18.7	92.0 ± 17.8	90.5 ± 17.0	90.7 ± 17.8	*p* < 0.0005 *	*p* = 0.38	*p* = 0.52
BMI (kg/m^2^)	24	30.4 ± 5.2			29.4 ± 5.2	23	31.1 ± 4.4			30.4 ± 4.0	*p* < 0.0005 *	*p* = 0.53	*p* = 0.57
WC (cm)	23	109.0 ± 13.9			98.2 ± 11.8	23	113.2 ± 12.3			105.5 ± 11.1	*p* < 0.0005 *	*p* = 0.10	*p* = 0.22
HC (cm)	23	117.2 ± 14.5			108.6 ± 10.4	23	117.0 ± 13.7			108.8 ± 8.1	*p* < 0.0005 *	*p* = 0.99	*p* = 0.92
WHR	23	0.9 ± 0.1			0.9 ± 0.1	23	1.0 ± 0.1			1.0 ± 0.1	*p* = 0.05 *	*p* = 0.02 *	*p* = 0.12
Body Fat (%)	22	36.5 ± 9.2	36.7 ± 9.6	35.3 ± 9.5	36.9 ± 11.3	17	36.3 ± 7.0	35.3 ± 7.7	35.0 ± 7.2	35.6 ± 7.7			
Muscle Mass (kg)	22	54.0 ± 11.7	52.7 ± 11.8	53.4 ± 11.4	51.8 ± 12.8	17	57.2 ± 13.3	56.9 ± 12.9	56.5 ± 13.3	56.0 ± 13.2	*p* < 0.0005 *	*p* = 0.36	*p* = 0.37
BPsys (mmHg)	23	124.0 ± 15.4			121.2 ± 14.3	23	137.8 ± 20.5			130.8 ± 16.2	*p* < 0.0005 *	*p* = 0.01 *	*p* = 0.30
BPdia (mmHg)	23	81.2 ± 11.7			77.9 ± 9.0	23	86.7 ± 9.8			83.8 ± 9.7	*p* < 0.0005 *	*p* = 0.04	*p* = 0.88

Values are presented as the mean ± SD. *n* = number of participants with complete data available for each outcome. *p* value obtained from a two-way mixed ANOVA test. * denotes significant difference, *p* < 0.05. BMI, body mass index; WC, waist circumference; HC, hip circumference; WHR, waist to hip ratio; BPsys, systolic blood pressure; BPdia, diastolic blood pressure.

**Table 3:** Primary outcome weight pre and post intervention.

	mRMR Group			eRMR Group					
	*n*	Baseline	Week 12	*n*	Baseline	Week 12	Time Effect, *p*	Group Effect, *p*	Time-by-Group Interaction, *p*
Weight (kg)	24	88.0 ± 13.7	85.2 ± 13.6	23	93.5 ± 17.4	91.4 ± 16.8	*p* < 0.0005 *	*p* = 0.20	*p* = 0.56

Values are presented as the mean ± SD. *n* = number of participants. *p* value obtained from a two-way mixed ANOVA test on pre and post data only. *denotes significant difference from baseline, *p* < 0.05.

**Table 4:** Biochemical outcomes at baseline and week 12.

	mRMR Group			eRMR Group					
	*n*	Baseline	Week 12	*n*	Baseline	Week 12	Time Effect, *p*	Group Effect, *p*	Time-by-Group Interaction, *p*
Glucose (mmol/L)	23	5.0 ± 0.7	4.7 ± 0.7	22	5.4 ± 0.9	5.1 ± 0.8	*p* < 0.0005 *	*p* = 0.08	*p* = 0.74
TC (mmol/L)	23	4.5 ± 1.1	4.4 ± 1.0	20	4.1 ± 0.7	4.2 ± 0.9	*p* = 0.92	*p* = 0.18	*p* = 0.45
HDL (mmol/L)	23	1.4 ± 0.4	1.3 ± 0.4	21	1.4 ± 0.4	1.3 ± 0.3	*p* < 0.0005 *	*p* = 0.91	*p* = 0.15
TC:HDL Ratio	23	3.4 ± 0.9	3.6 ± 0.9	19	3.0 ± 0.6	3.2 ± 0.8	*p* = 0.09	*p* = 0.07	*p* = 0.87
LDL (mmol/L)	19	2.7 ± 0.7	2.6 ± 0.8	17	2.0 ± 0.6	2.3 ± 0.8	*p* = 0.43	*p* = 0.02 *	*p* = 0.28
TG (mmol/L)	20	1.2 ± 0.5	1.1 ± 0.4	18	1.3 ± 0.4	1.2 ± 0.4	*p* = 0.16	*p* = 0.63	*p* = 0.67

Values are presented as the mean ± SD. *n* = number of participants with complete data available for each outcome. *p* value obtained from a two-way mixed ANOVA test. TC, total cholesterol; HDL, high-density lipoprotein; TC:HDL, total cholesterol to high-density lipoprotein ratio; LDL, low-density lipoprotein; TG, triglycerides. *denotes significant difference, *p* < 0.05.

**Table 5:** Metabolic outcomes and estimated energy intake for groups across the intervention period.

Variable	mRMR Group					eRMR Group							
	*n*	Baseline	Week 3	Week 6	Week 12	*n*	Baseline	Week 3	Week 6	Week 12	Time Effect, *p*	Group Effect, *p*	Time-by-Group Interaction, *p*
mRMR (kcal)	22	1764.3 ± 547.6	1687.7 ± 505.8	1737.8 ± 475.1	1688.3 ± 454.6	16	1813.8 ± 410.4	1688.8 ± 469.6	1787.4 ± 566.5	1780.5 ± 518.8	*p* = 0.22	*p* = 0.38	*p* = 0.75
RER	22	0.8 ± 0.2	0.8 ± 0.1	0.8 ± 0.1	0.8 ± 0.1	16	0.8 ± 0.1	0.8 ± 0.1	0.8 ± 0.1	0.8 ± 0.1	*p* = 0.32	*p* = 0.75	*p* = 0.34
eRMR (kcal)	22	1578.5 ± 227.7	1576.0 ± 230.9	1553.7 ± 222.5	1547.0 ± 225.0	17	1656.9 ± 297.7	1648.4 ± 276.4	1624.1 ± 282.2	1625.5 ± 288.0	*p* < 0.0005 *	*p* = 0.37	*p* = 0.79
eEI (kcal)	15	2327.3 ± 827.1			1841.1 ± 534.3	15	2117.3 ± 562.8			1645.1 ± 433.3	*p* < 0.0005 *	*p* = 0.28	*p* = 0.96

Values are presented as the mean ± SD. *n* = number of participants with complete data set available for each variable. *p* value obtained from a two-way mixed ANOVA test. * denotes significant difference, *p* < 0.05. mRMR, measured resting metabolic rate; RER, respiratory exchange ratio; eRMR, estimated resting metabolic rate (Mifflin-St. Jeor equation); eEI, estimated energy intake (group analysis from a 3-day food diary).

**Table 6:** Estimated energy intake at baseline and week 12 for male and female participants.

	Intervention Group	Sex	*n*	Baseline	Week 12	Sex	*n*	Baseline	Week 12
eEI (kcal)	mRMR	M	5	2462 ± 973	2052 ± 597	F	10	2260 ± 793	1736 ± 473
	eRMR	M	8	2267 ± 724	1804 ± 445	F	15	1947 ± 253	1463 ± 367

Values are presented as the mean ± SD. *n* = number of participants with complete data available at each time point. eEI, estimated energy intake (from a a3 day food diary) for male and female participants.

**Table 7:** Prescribed energy intake versus estimated energy intake at week 12 for male and female participants.

	Intervention Group	Sex	*n*	Prescribed	Estimated Week 12	Sex	*n*	Prescribed	Estimated Week 12
Energy Intake (kcal)	mRMR	M	5	2180 ± 253	2052 ± 597	F	10	1584 ± 271	1736 ± 473
	eRMR	M	8	2100 ± 236	1804 ± 445	F	15	1500 ± 141	1463 ± 367

Values are presented as the mean ± standard deviation. *n* = number of participants with complete data set available at each time point. Estimated 12-week daily average energy intake from a 3-day food diaries.

## Data Availability

The study was conducted in accordance with the Data Protection ACT, 2018 and approved by the Institute’s Data Protection Officer. Researchers seeking the analysis dataset for this work should submit requests to the corresponding author.

## Supplementary Materials

The following are available online at https://www.mdpi.com/article/10.3390/nu13041229/s1. Supplementary Figure S1 Informed consent was obtained from all subjects involved in the study
