# Supplementary material for: Evaluation of Measured Resting Metabolic Rate for Dietary Prescription in Ageing Adults with Overweight and Adiposity-Based Chronic Disease
Source: Nutrients. 2021 Apr 8;13(4):1229. doi: 10.3390/nu13041229 (PMC8068182; doi:10.3390/nu13041229)
Supplement: Supplementary file 1 [file nutrients-13-01229-s001.pdf]

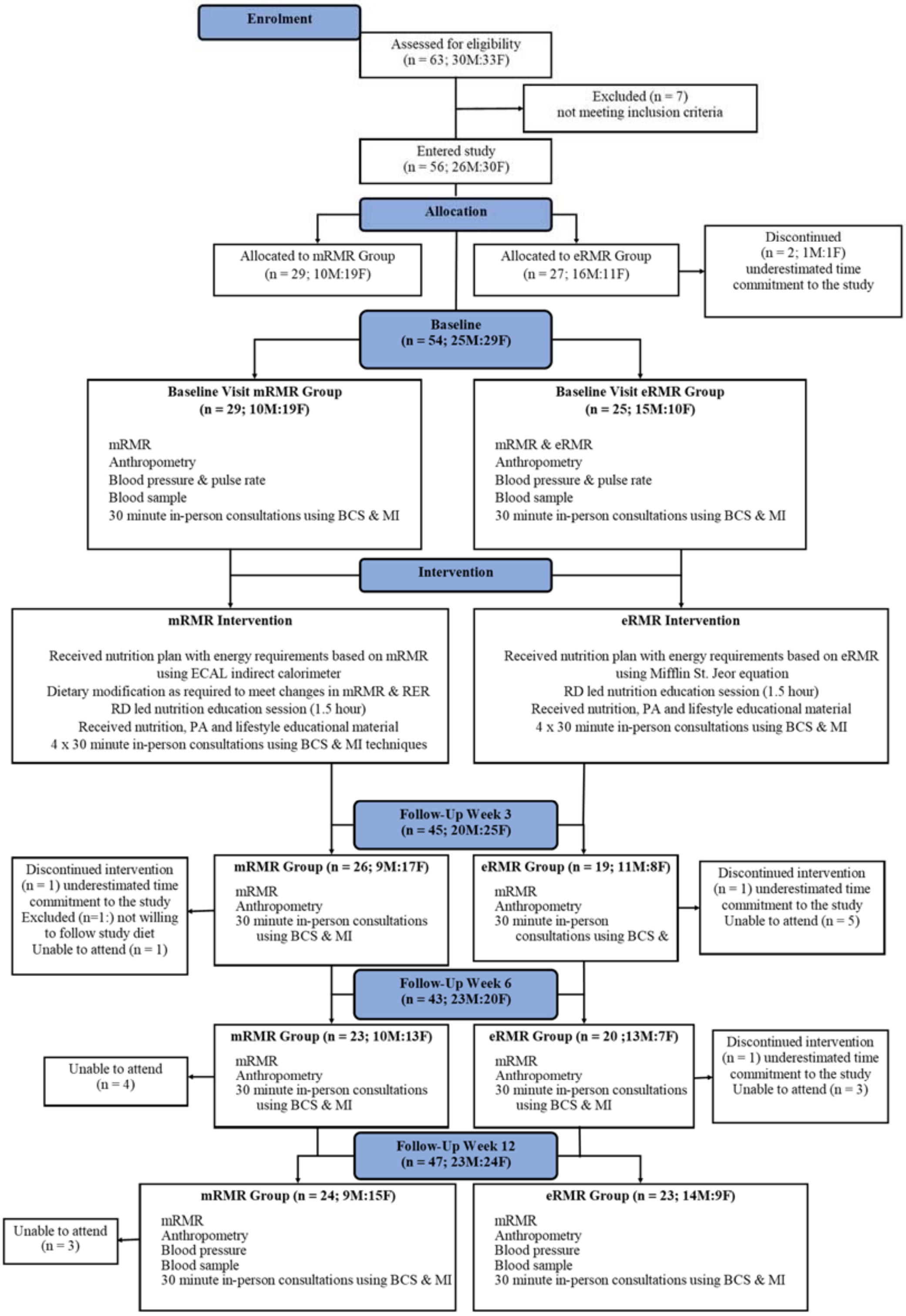

Online Resource 1. Participant and study flow diagram

Ciara Cooney [1], Lisa Ryan [1,*], Maria McDonagh [1]

1 Department of Natural Sciences, School of Science and Computing, Galway-Mayo Institute of Technology, Galway, Ireland; ciara.cooney@gmit.ie, lisa.ryan@gmit.ie, Maria.McDonagh@gmit.ie

* Correspondence: Lisa Ryan, Head of Department of Natural Sciences, School of Science and Computing, Galway-Mayo Institute of Technology, Dublin Road, Galway, Ireland; lisa.ryan@gmit.ie; Tel.: +353 (0)91 742556
